# Tele-ECG consulting and outcomes on primary care patients in a low-to-middle income population: the first experience from Makassar telemedicine program, Indonesia

**DOI:** 10.1186/s12875-020-01325-4

**Published:** 2020-11-30

**Authors:** Idar Mappangara, Andriany Qanitha, Cuno S. P. M. Uiterwaal, Jose P. S. Henriques, Bastianus A. J. M. de Mol

**Affiliations:** 1 Department of Cardiology and Vascular Medicine, Faculty of Medicine, Hasanuddin University, Makassar, Indonesia; 2 Department of Physiology, Faculty of Medicine, Hasanuddin University, Makassar, Indonesia; 3 Department of Cardio-thoracic Surgery, Amsterdam University Medical Center, Amsterdam, The Netherlands; 4 Julius Global Health, Julius Center for Health Sciences and Primary Care, University Medical Center Utrecht, Utrecht, The Netherlands; 5 Department of Cardiology, Amsterdam University Medical Center, Amsterdam, The Netherlands

**Keywords:** Telemedicine, Tele-ECG, Primary care, Low- and middle-income country, Quality of care, Pre-hospital triage

## Abstract

**Background:**

Telemedicine has been a popular tool to overcome the lack of access to healthcare facilities, primarily in underprivileged populations. We aimed to describe and assess the implementation of a tele-electrocardiography (ECG) program in primary care settings in Indonesia, and subsequently examine the short- and mid-term outcomes of patients who have received tele-ECG consultations.

**Methods:**

ECG recordings from thirty primary care centers were transmitted to Makassar Cardiac Center, Indonesia from January to July 2017. We cross-sectionally measured the performance of this tele-ECG program, and prospectively sent a detailed questionnaire to general practitioners (GPs) at the primary care centers. We performed follow-up at 30 days and at the end of the study period to assess the patient outcomes.

**Results:**

Of 505 recordings, all (100%) ECGs were qualified for analysis, and about half showed normal findings. The mean age of participants was 53.3 ± 13.6 years, and 40.2% were male. Most (373, 73.9%) of these primary care patients exhibited manifested CVD symptom with at least one risk factor. Male patients had more ischemic ECGs compared to women (*p* < 0.01), while older age (> 55 years) was associated with ischemic or arrhythmic ECGs (*p* < 0.05). Factors significantly associated with a normal ECG were younger age, female gender, lower blood pressure and heart rate, and no history of previous cardiovascular disease (CVD) or medication. More patients with an abnormal ECG had a history of hypertension, known diabetes, and were current smokers (*p* < 0.05). Of all tele-consultations, GPs reported 95% of satisfaction rate, and 296 (58.6%) used tele-ECG for an expert opinion. Over the total follow-up (14 ± 6.6 months), seven (1.4%) patients died and 96 (19.0%) were hospitalized for CVD. Of 88 patients for whom hospital admission was advised, 72 (81.8%) were immediately referred within 48 h following the tele-ECG consultation.

**Conclusions:**

Tele-ECG can be implemented in Indonesian primary care settings with limited resources and may assist GPs in immediate triage, resulting in a higher rate of early hospitalization for indicated patients.

**Supplementary Information:**

The online version contains supplementary material available at 10.1186/s12875-020-01325-4.

## Background

To date, telemedicine has become a popular tool in overcoming geographical barriers and increasing access to healthcare services. This particularly benefits the rural and underserved populations in low- and middle-income countries – groups that traditionally suffer from lack of access to healthcare [1]. The World Health Organization has defined telemedicine as the delivery of healthcare services, where distance is a critical factor, using information and communication technologies for the exchange of valid information for the diagnosis, treatment, and prevention of disease, for research and for evaluation [1].

Indonesia is the worlds’ largest archipelago and the most populated nation in South-East Asia; it consists of 17,508 islands and has a population of more than 260 million people [2]. More than half of the Indonesian population lives in Java, with the rest distributed unevenly across ~ 6000 islands [2, 3]. Of this population, > 10% live in poverty [4]. Cardiovascular disease (CVD) is the leading cause of death in this lower middle-income country, responsible for ~ 37% of total deaths [4]. Premature deaths from coronary artery disease (CAD), stroke, and diabetes are significantly higher in Indonesia compared to neighboring countries [4]. In 2016, the latest analyses of the Global Burden of Disease reported that these diseases are also the top three causes of disability-adjusted life-years (DALYs) in Indonesia [2]. Despite the high burden of CVD in this nation, in 2016 only 1.5 cardiologists per 1,000,000 population were available [4], and in 2013 there were ~ 30 cardiac centers (half located in Java) to serve > 2.6 million prevalent cases of CAD [5, 6].

In view of the shortage of cardiologists and evident demand for expertise in cardiovascular care, Makassar Cardiac Center initiated the first telemedicine project in Eastern Indonesia – transferring the electrocardiography (ECG) recordings from primary care facilities to a center of expertise at Hasanuddin University Hospital. This project entailed decision support for general practitioners (GPs) in primary care when confronted with patients with symptoms or risk factors for CVD. Although the implementation of telemedicine program has started in Indonesia since 2012, reports on the performance and outcomes of the program remain less explored.

We aimed [1]: to study in detail the implementation of the tele-ECG program in primary care settings of a South-East Asian population with limited resources; and [2] to assess the short- and mid-term consequences of patient outcomes in relation to decision-making assisted by this tele-ECG consultation. To this end, we conducted a population-based cohort study in primary care patients in Makassar, Indonesia.

## Methods

### The tele-ECG program

**Fig. 1:**
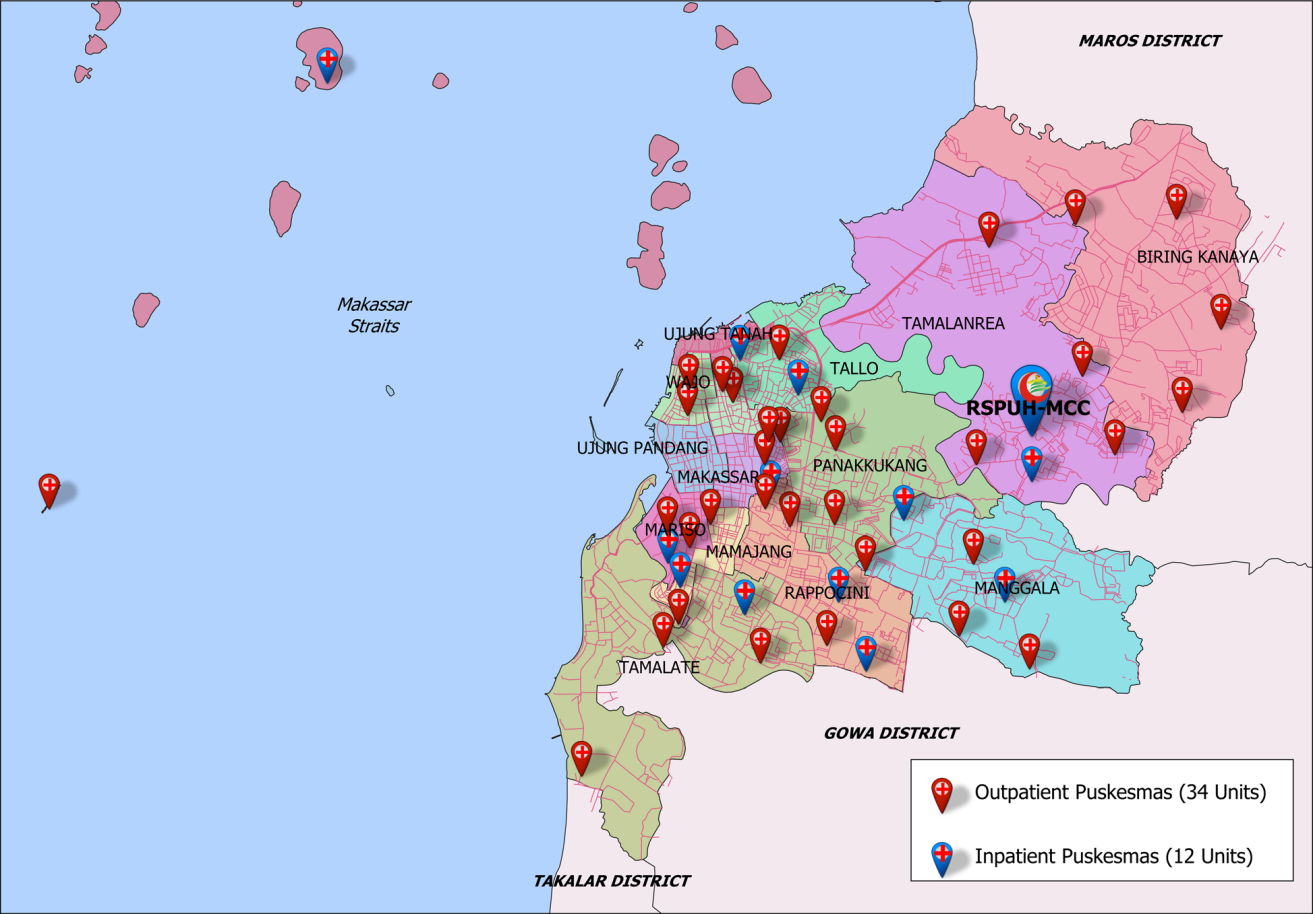
Primary care centers (Puskesmas) in Makassar City (199.3 km^2^). RSPUH = Rumah Sakit Pendidikan Universitas Hasanuddin (Hasanuddin University Hospital); MCC = Makassar Cardiac Center. Source: https://tanahair.indonesia.go.id/portal-web/download/perwilayah (freely usable)

In the initiation of this telemedicine program, Makassar Cardiac Center in collaboration with the local government of Makassar City provided one digital ECG machine for each primary care center in 2015. Distribution of all primary care centers in the city of Makassar is depicted in Fig. 1.

### Study population

**Fig. 2:**
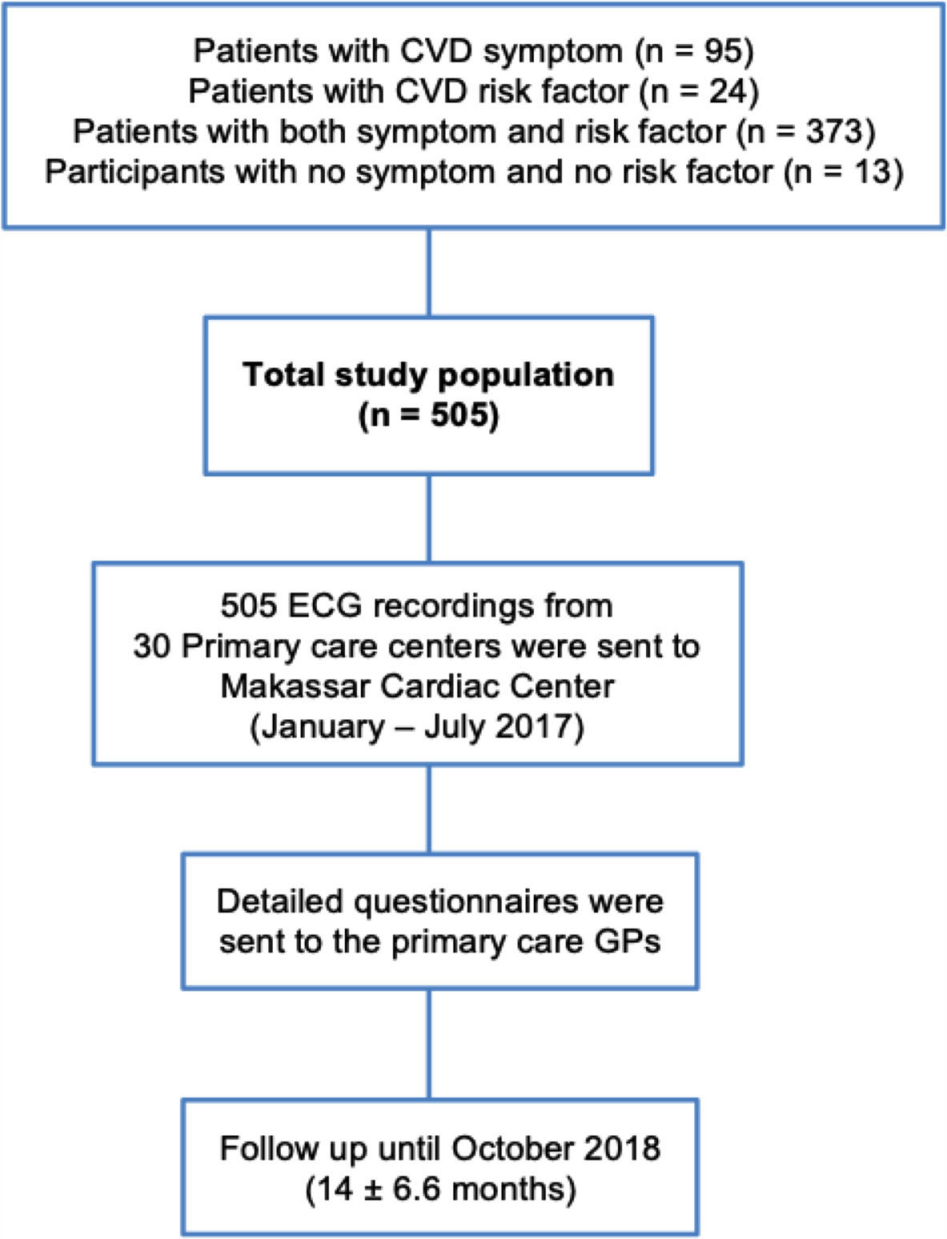
Flowchart of the study population. ECG = electrocardiogram; Puskesmas = Pusat Kesehatan Masyarakat; GP = General Practitioner

Between January and July 2017, 12-lead ECG recordings from thirty primary care centers (known as Pusat Kesehatan Masyarakat or Puskesmas) were transmitted to Hasanuddin University Hospital. We prospectively collected data from patient medical records and conducted interviews by sending a questionnaire to primary care GPs. Figure 2 shows the flowchart of the present study.

Patients were eligible for ECG assessment if they presented to the Puskesmas with a cardiovascular symptom (*n* = 95), risk factor (*n* = 24), or both (*n* = 373). Patients with other diseases or healthy subjects (*n* = 13) who voluntarily asked to have an ECG examination were also enrolled. None of emergency cases that required urgent hospital admission, nor patients who refused to sign informed consent were reported during this study recruitment.

### Data collection and measurement

A detailed questionnaire was designed to obtain data on socio-demographic and clinical profiles (i.e. symptoms, onset, prior diseases, prior medications, anthropometric status, vital signs, and cardiovascular risk factors: hypertension, diabetes mellitus, current smoking, and family history of CVD), management and medications after tele-ECG, and GP’s reasons and satisfaction with tele-ECG consulting.

Vital sign measurements (i.e. blood pressure, heart rate, respiration rate, and axillary temperature), anthropometrics, standard physical examination, and ECG assessment were performed on all participants. Body weight, height, and waist circumference were measured manually. No laboratory tests (e.g. fasting plasma glucose, lipid profiles, and creatinine) were performed, as these tests are generally not available at the primary care level in Makassar.

All ECG assessments were performed by trained primary care GPs or nurses following standard guidelines and protocols, using an automated ECG machine (BTL-08 SD ECG, BTL Industries Ltd., Hertfordshire, UK). The ECG files were sent through the internet to the analysis service center at Hasanuddin University Hospital and saved in the hospital database. Two cardiologists independently reviewed and analyzed each ECG recording, and mutually agreed to all the interpretations. The implementation of tele-ECG consultation in a primary care center and illustration of ECG in Makassar Medical System are described in Figure S1.

### Definitions and classification

A CVD symptom was defined as either mild-to-moderate chest pain (angina), shortness of breath (dyspnea), palpitations, heartburn, lightheadedness (dizziness), headache, or syncope. Hypertension, diabetes mellitus, current smoking, family history of CVD, and obesity were categorized as the risk factors.

We classified the participants based on their ECG findings into normal and abnormal ECG categories. We used a hierarchical manner to determine the classification of the ECG patterns. The order of the categorization was ischemia, arrhythmia, structural change, and others, respectively. Management after tele-ECG was classified as: referral to the hospital, outpatient with no medications, and outpatient with cardiovascular medications for primary or secondary prevention.

### ECG assessment and referral

The ECGs sent to the service center were analyzed daily. The advice for referral was based on the ECG findings and the severity of the symptoms presented. Patients were referred if they had a marked CVD symptoms and the ECG showed an abnormal finding. Criteria for referral were patients with angina and ischemic ECG; patients with dyspnea and ischemic or structural-related ECG; and patients with palpitations, syncope or other symptoms with arrhythmic ECG. Where criteria were met or if there was doubt about the ECG, the advice was to refer the patient to a hospital with cardiovascular care facilities. GPs made the final decision on the urgency of the referral based on their own assessment. Once referred, the patient came under the responsibility of the cardiologists for diagnostic work-up and treatment.

### Follow-up and outcomes

After ECG assessment, we followed the patients and measured the adverse outcomes (i.e. cardiovascular death and hospitalization) at 30 days and at the end of the study period, up to 30 October 2018. Cause of death was determined largely based on family reports. We defined as a CVD death if patients died suddenly at home, on the way to the hospital, or during hospitalization due to CVD; or had shown a prior cardiovascular symptom or sign, resulting in an abrupt death. Whereas CVD hospitalization was defined as being hospitalized due to ischemic heart disease, heart failure, or stroke. Primary care nurses, cadres, and research assistants performed the follow-up and obtained data from the primary care medical records, also via telephone calls, or home visits. None of the participants were lost to follow-up.

### Statistical analysis

For continuous variables, means ± standard deviations (SD) were calculated, while categorical variables were expressed as a proportion (percentage). Median (Q1-Q3) was used for the skewed data. Differences in continuous variables were estimated using the t-test for independent samples or Mann-Whitney U test. Proportions were compared using Pearson’s Chi-square or Fisher’s Exact tests. Baseline and clinical profiles, CVD symptoms and risk factors, management in primary care, and GP reason and satisfaction on tele-ECG were presented in accordance with the ECG conclusion (normal vs. abnormal ECG). The rates of cardiovascular death and hospitalization at 30 days and >  30 days until the end of follow-up were compared in the referral (abnormal ECG) vs. non-referral (normal and abnormal ECG) groups. A two-tailed *p*-value < 0.05 was considered statistically significant. Data management and statistical computation were performed with IBM SPSS Ver. twenty-three for Mac.

## Results

From January to July 2017, a total of 505 ECG recordings were received in the analysis center of the telemedicine program, at Hasanuddin University Hospital. All ECG recordings qualified for analysis. The mean age of participants was 53.3 ± 13.6 years, and 203 (40.2%) were male. We classified 253 (50.1%) participants into normal, and 252 (49.9%) into abnormal ECG groups.

**Fig. 3:**
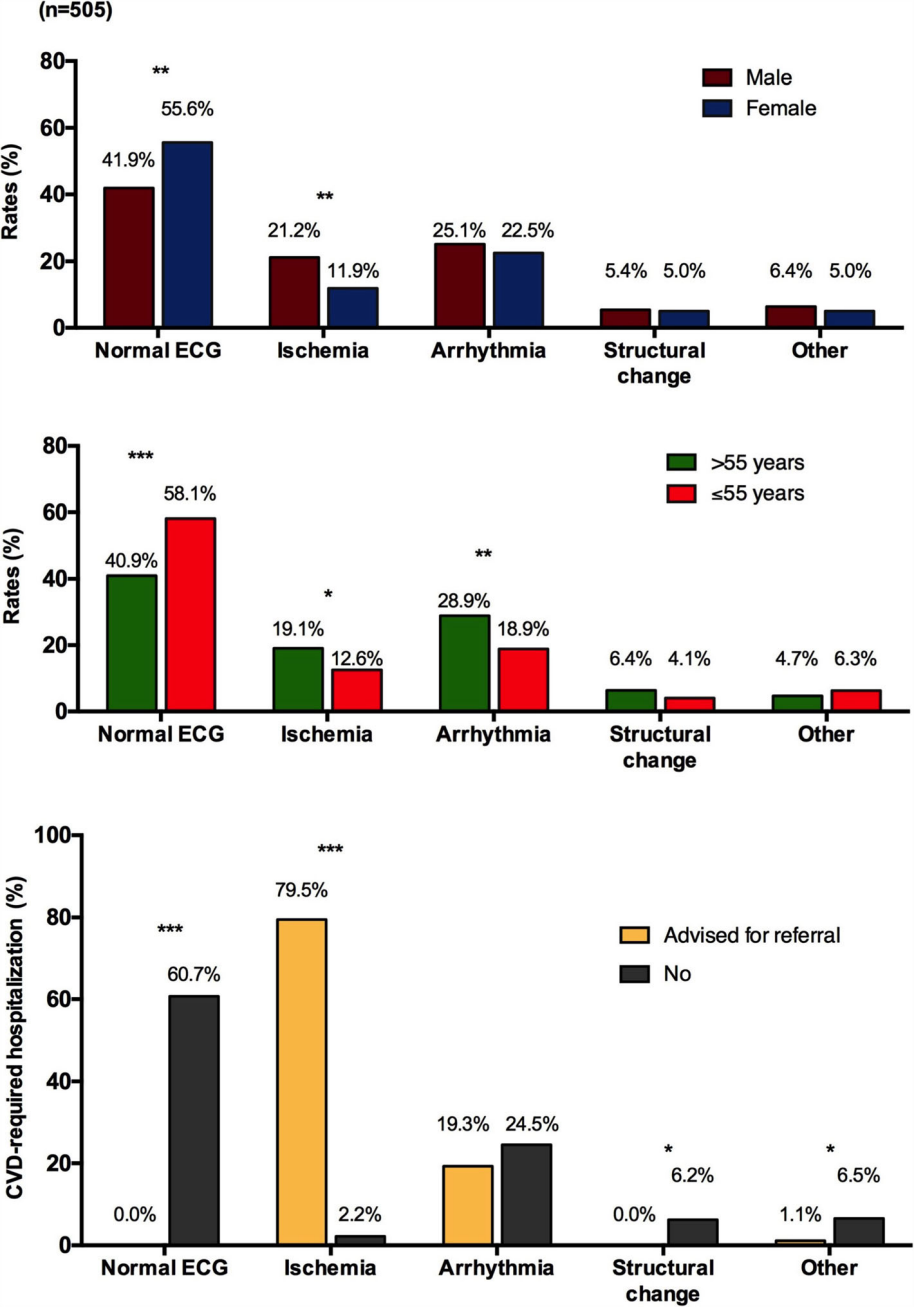
Distribution of ECG findings in the study population according to age, sex, and advice for referral. *p < 0.05; **p < 0.01; ***p < 0.001

In Table 1, we present the baseline and clinical profiles of the study population according to ECG classification. Patients with a normal ECG were significantly younger, mostly female, had lower systolic and diastolic blood pressure as well as a lower heart rate, and fewer had previously suffered from CVD or taken cardiovascular medications, compared to those with abnormal ECG findings. Of all, 414 (82.0%) participants were of low and middle socio-economic status. More men than women were prone to ischemic ECG (*p* < 0.01), while older age (> 55 years) was associated with an ischemic or arrhythmic pattern (*p* < 0.05) **(**see Fig. 3**)**.

**Table 1:** Baseline and clinical characteristics of the participants according to ECG findings

Variables	Normal ECG (***n*** = 253)	Abnormal ECG (***n*** = 252)	Total (***n*** = 505)	***p***-value
Age (years)	50.7 ± 14.1	56.0 ± 12.6	53.3 ± 13.6	< 0.001
Male sex	85 (33.6)	118 (46.8)	203 (40.2)	0.002
Systolic BP (mmHg)	124.7 ± 15.6	136.2 ± 22.2	130.4 ± 20.0	< 0.001
Diastolic BP (mmHg)	79.5 ± 8.2	82.5 ± 9.4	81.0 ± 8.9	< 0.001
Heart rate (bpm)	77.9 ± 9.5	83.5 ± 16.9	80.7 ± 14.0	< 0.001
BMI (kg/m^2^)^a^	24.2 (21.8–26.7)	24.1 (21.4–27.2)	24.2 (21.6–26.9)	0.788
Low-to-middle SES	213 (84.2)	201 (79.8)	414 (82.0)	0.196
**Previous Diseases:**				
Cardiovascular disease^b^	2 (0.8)	8 (3.2)	10 (2.0)	< 0.001
COPD^b^	5 (2.0)	2 (0.8)	7 (1.4)	0.055
**Previous Medications:**				
Anti-hypertension	61 (24.1)	110 (43.7)	171 (33.9)	< 0.001
Anti-diabetic	14 (5.5)	29 (11.5)	43 (8.5)	0.016
Anti-cholesterol	9 (3.6)	19 (7.5)	28 (5.5)	0.051
Anti-platelet	1 (0.4)	11 (4.4)	12 (2.4)	0.003
Anti-arrhythmia^b^	0 (0.0)	2 (0.8)	2 (0.4)	0.249

Values are n (%) or means ± SD, unless otherwise stated. Comparison was performed using independent-samples t-test for continuous variables and Pearson Chi-square test for categorical variables

*ECG* electrocardiogram, *BP* blood pressure, *bpm* beat per minute, *BMI* body mass index, *SES* socio-economic status, *COPD* chronic obstructive pulmonary disease

^a^Values are medians (Q1-Q3). Comparison was done using Mann-Whitney U test

^b^Comparison was performed using Fisher’s Exact test

The CVD symptoms and risk factors of the primary care patients are shown in Table 2. More female patients with chest pain had a normal ECG (*p* = 0.01). A longer duration of angina (≥ 15 min) and marked dyspnea were associated with an abnormal ECG (*p* = 0.045 and *p* = 0.001, respectively). More patients with abnormal ECG had a history of hypertension, known diabetes, and were current smokers (*p* < 0.05). Half of the participants (254, 50.3%) had hypertension, and 171 (67.3%) were on medication. Of all participants, 214 (42.4%) were obese.

**Table 2:** CVD symptoms and risk factors of the cohort based on ECG recordings

Variables^**a**^	Normal ECG (***n*** = 253)	Abnormal ECG (***n*** = 252)	Total (***n*** = 505)	***p***-value
**CVD Symptoms:**				
Chest pain	164 (64.8)	153 (60.7)	317 (62.8)	0.340
≥ 15 min.	17 (6.7)	30 (11.9)	47 (9.3)	0.045
Female sex	105 (64.0)	76 (49.7)	181 (57.1)	0.010
Heartburn	19 (7.5)	14 (5.6)	33 (6.5)	0.374
Dyspnea	27 (10.7)	54 (21.4)	81 (16.0)	0.001
Palpitation	24 (9.5)	38 (15.1)	62 (12.3)	0.055
Syncope^b^	0 (0.0)	1 (0.4)	1 (0.2)	0.499
Dizziness/headache	20 (7.9)	24 (9.5)	44 (8.7)	0.519
Recurrent symptoms	64 (25.3)	78 (31.0)	142 (28.1)	0.157
**CVD Risk Factors:**				
Hypertension	99 (39.1)	155 (61.5)	254 (50.3)	< 0.001
Known diabetes	15 (5.9)	29 (11.5)	44 (8.7)	0.026
Current smoking	37 (14.6)	70 (27.8)	107 (21.2)	< 0.001
Sticks/day	10 ± 7	12 ± 6	11 ± 6	0.147
Family CVD	15 (5.9)	12 (4.8)	27 (5.3)	0.067
Obese (BMI ≥25)	106 (41.9)	108 (42.9)	214 (42.4)	0.827

Values are n (%) or means ± SD, unless otherwise stated. Comparison was performed using independent-samples t-test for continuous variables and Pearson Chi-square test for categorical variables

*ECG* electrocardiogram, *CVD* cardiovascular disease, *min*. minutes

^a^More than one symptom and/or risk factor is possible

^b^Comparison was performed using Fisher’s Exact test

Profile of the participants and advice for referral are described in Table 3. Overall, 79 (15.6%) patients were categorized as having ischemia, 119 (23.6%) as having arrhythmia, and 26 (5.1%) had structural changes. The majority of participants (373, 73.9%) presented to the primary care center with manifested CVD symptoms and with at least one risk factor, while 37 (7.3%) were asymptomatic. Of 88 patients for whom hospital admission was advised, 70 (79.5%) showed ischemic ECG (Fig. 3).

**Table 3:** Patient profiles and advice for hospital admission based on ECG findings

Patient profiles	Normal ECG (***n*** = 253)	Abnormal ECG^**a**^					***p***-value
		Ischemia (***n*** = 79)	Arrhythmia (***n*** = 119)	Structural changes (***n*** = 26)	Others (***n*** = 28)	Advice for hospital admission (***n*** = 88)	
Symptom only	55 (21.7)	8 (10.1)	21 (17.6)	6 (23.1)	5 (17.9)	9 (10.2)	0.218
Symptom (+) with 1 risk factor	119 (47.0)	21(26.6)	57 (47.9)	8 (30.8)	11 (39.3)	27 (30.7)	0.010
Symptom (+) with > 1 risk factors	51 (20.2)	46 (58.2)	39 (32.8)	11 (42.3)	10 (35.7)	48 (54.5)	< 0.001
Risk factors only	18 (7.1)	3 (3.8)	1 (0.8)	1 (3.8)	1 (3.6)	3 (3.4)	0.115
No symptom and no risk factor	10 (4.0)	1 (1.3)	1 (0.8)	0 (0.0)	1 (3.6)	1 (1.1)	0.322

Values are n (%). Comparison was performed using Pearson Chi-square test

*ECG* electrocardiogram

^a^Categorization based on dominated pattern presented on ECG

Table 4 presents the GPs reasons, satisfaction, and management after tele-ECG consultation. The majority of the GPs carried out a consultation through tele-ECG for expert opinion (296, 58.6%) and because of manifested or moderate CVD symptoms (192, 38.0%). Overall, 154 (30.5%) patients were observed in Puskesmas for primary or secondary prevention with adequate medications; while 88 (34.9%) patients with abnormal ECG were referred to the hospital. Of all tele-ECG consultations, the primary care GPs reported an ~ 95% of satisfaction rate.

**Table 4:** GP’s reason, management, and satisfaction on tele-ECG consulting

Variables	Normal ECG (***n*** = 253)	Abnormal ECG (***n*** = 252)	Total (***n*** = 505)	***p***-value
**GP’s reason for tele-ECG:**				
Manifested CVD symptoms	76 (30.0)	116 (46.0)	192 (38.0)	< 0.001
Unable to interpret the ECG	2 (0.8)	12 (4.8)	14 (2.8)	0.007
Ask for an expert opinion	175 (69.2)	121 (48.0)	296 (58.6)	< 0.001
Others^a^	0 (0.0)	3 (1.2)	3 (0.6)	0.124
**Management after tele-ECG:**				
Refer to hospital	0 (0.0)	88 (34.9)	88 (17.4)	< 0.001
Outpatient without medications	183 (72.3)	80 (31.7)	263 (52.1)	< 0.001
Outpatient with new or continued medications	70 (27.7)	84 (33.3)	154 (30.5)	0.167
**Medications at primary care following tele-ECG consultation**:				
Aspirin	2 (0.8)	49 (19.4)	51 (10.1)	< 0.001
Clopidogrel^a^	0 (0.0)	8 (3.2)	8 (1.6)	0.004
Beta blocker^a^	0 (0.0)	8 (3.2)	8 (1.6)	0.004
Calcium-channel blocker	53 (20.9)	95 (37.7)	148 (29.3)	< 0.001
ACE inhibitor	7 (2.8)	54 (21.4)	61 (12.1)	< 0.001
Angiotensin receptor blocker	4 (1.6)	10 (4.0)	14 (2.8)	0.102
Diuretic	0 (0.0)	17 (6.7)	17 (3.4)	< 0.001
Nitrate	13 (5.1)	47 (18.7)	60 (11.9)	< 0.001
Lipid-lowering agents	8 (3.2)	39 (15.5)	47 (9.3)	< 0.001
**GP’s satisfaction on tele-ECG:**				
Yes	232 (91.7)	247 (98.0)	479 (94.9)	0.001

Values are n (%) or mean. Comparison was performed using Pearson Chi-square test

*ECG* electrocardiogram, *CVD* cardiovascular disease

^a^Comparison using Fisher’s Exact test. The study design required the GPs to send all ECG assessments to the database center

Over the entire follow-up period (14 ± 6.6 months), seven (1.4%) patients died, and 96 (19.0%) were admitted to the hospital for CVD. Table 5 compares the adverse outcomes between abnormal vs. normal ECG groups. Eighty-eight (34.9%) patients with abnormal ECG were advised for hospital admission; of those, 72 (81.8%) were sent immediately to the hospital within 48 h following consultation. Over the 30 days, there were no significant differences between patients with abnormal and normal ECG in terms of mid-term cardiovascular death (2.0% vs. 0.4%, *p* = 0.122) and hospitalization (3.2% vs. 2.8, *p* = 0.800).

**Table 5:** Major adverse cardiovascular events (MACE) in normal and abnormal ECG groups

MACE	Abnormal ECG		Normal ECG	***p***-value^**a**^
	Referred (***n*** = 88)	Not referred (***n*** = 164)	Not referred (***n*** = 253)	
**≤ 30 days**				
CVD death	0 (0.0)	0 (0.0)	1 (0.4)	1.000
CVD hospitalization	75 (85.2)	6 (3.7)	0 (0.0)	< 0.001
**Admission within 48 h**	**72 (81.8)**	**5 (3.0)**	**0 (0.0)**	**< 0.001**
Total	75 (85.2)	6 (3.7)	1 (0.4)	< 0.001
**> 30 days**				
CVD death	2 (2.3)	3 (1.8)	1 (0.4)	0.122
CVD hospitalization	2 (2.3)	6 (3.7)	7 (2.8)	0.800
Total	4 (4.5)	9 (5.5)	8 (3.2)	0.276

Values are n (%). Comparison was performed using Pearson Chi-square and Fisher’s Exact test

*ECG* electrocardiogram, *CVD* cardiovascular disease, *min*. minutes

^a^Comparisons were done between abnormal vs. normal ECG groups

## Discussion

This study shows that tele-ECG consulting in a low-to-middle income Indonesian population was helpful to support primary care GPs in making a quick pre-hospital triage. Of 505 ECG screenings transmitted to the analysis center, all recordings were qualified for analysis. Within 30 days, tele-ECG is associated with a higher rate of early hospitalization when needed. We found no significant differences between the normal and abnormal ECG groups in terms of mid-term cardiovascular death and hospitalization.

From our analyses, we found that patients with a normal ECG were predominantly female, younger, showed better clinical profiles, and had fewer CVD risk factors when compared to those with an abnormal ECG. Men were significantly more prone to have an ischemic ECG than women, and participants of older age (> 55 years) were more susceptible to ischemic or arrhythmic ECG compared with those of younger age groups.

Half of the participants in this study suffered from hypertension; 22% were unaware of this and 33% were untreated. The National Survey 2013 reported that 62% of hypertension cases in the Indonesian general population were undiagnosed [6]. A previous review also reported that > 50% of the study participants with hypertension in Indonesia were unaware and untreated [4]. This recent study showed a lower number of unaware and untreated cases, as the majority of our study population turned up to a primary care center with cardiovascular symptoms and risk factors. However, these numbers are still higher compared with the 16% unaware and 7% untreated hypertension cases in stroke patients recently studied in China [7, 8].

A large proportion of the population in Indonesia is estimated to have undiagnosed diabetes, and diabetes is often detected only once patients present with secondary complications [4]. Nevertheless, standard screening and detection for diabetes mellitus and dyslipidemia (i.e. fasting plasma glucose and lipid profiles) are usually unavailable at primary care services in this country. In our study, ~ 9% of participants had known diabetes. We inferred that considering the moderate-to-high risk profile there must be more undetected or undiagnosed diabetes cases in this study population.

From the present study, we observed a higher number of female patients who presented with chest pain despite a normal ECG. There is abundant evidence to indicate that women are more likely to present with chest pain – and often with recurrence and re-admissions – compared to men [9]. However, CAD occurs more frequently in men [9]. Another study also indicated that women scored the intensity of their chest pain significantly higher than men [10]. Non-CAD-related angina is commonly associated with persistent chest pain, causing poor function and quality of life, and re-admission [9]. Therefore, in women with a normal ECG, it should be kept in mind that if the symptom is moderate and recurring, the angina should not be underestimated. Microvascular dysfunction, coronary artery spasm, coronary artery dissection, and myocardial bridging are the most common causes of chest pain in women who present at the Emergency Department [9]. These underlying patho-mechanisms may be undetectable on a one-time point resting ECG assessment. Women are more vulnerable to longer admission to the hospital, slower diagnosis, and inadequate treatment [11]. Previous studies have suggested that coronary angiography is used less often in women, largely because their risk is underestimated [11]. Women describe an atypical clinical feature of chest pain, which significantly differs from men. Often, women complain of concomitant atypical symptoms (e.g. heartburn to epigastric pain, unusual fatigue, dizziness, feeling of doom, and generalized weakness) [9], and make the indication for CVD even more difficult to establish.

Symptomatic patients with normal ECG findings are often reassured by their diagnosis and favorable prognosis, but receive no specific prevention management, despite the presence of a higher risk of CVD events. Despite the moderate-to-high risk profiles, ~ 52% of our study population received no medications, while ~ 31% received adequate medications and planned for long-term primary or secondary prevention.

In this study, we focused on evaluating the qualitative interpretation of tele-ECG performance, quantifying patient profiles and management, and conducting an in-depth case analysis of all deaths and hospitalizations observed during the first 30 days and >  30 days after tele-ECG advice. Based on the patient risk profile and clinical history, we obtained a reasonable picture regarding the quality of care and the impact of the tele-ECG consultation. In the non-referral group with abnormal ECG, six (3.7%) patients had been hospitalized for CVD within 30 days, three (1.8%) patients died, and 6 (3.7%) were admitted to the hospital after 30 days. While in the normal ECG group, two (0.8%) patients died due to uncontrolled diabetes and heart failure, while 7 (2.8%) were admitted to the hospital due to CVD during the follow-up period. This indicates that the criteria for referral should be revised, and patients with recurrent and marked cardiovascular symptoms should be treated with caution despite their normal ECG presentation. In the future, GP decisions should be supported by reliable and standardized scores and algorithms, which are currently not available in primary care services.

At mid-term follow-up, there were no significant differences between the referral and non-referral groups pertaining to cardiovascular death and hospitalization. We can assume that [1]: the low cardiovascular mortality rate in the abnormal ECG group indicated that early hospitalization based on tele-ECG advice had a favorable impact [2]; the higher rate of CVD hospitalization in the normal ECG group indicated that those patients could have undetectable and uncontrolled cardiovascular risk factors, particularly because standard screening for diabetes and dyslipidemia is generally not available in primary care centers in Indonesia; and [3] the lower rate of mid-term CVD hospitalization implied well-controlled or prevention of CVD risk factors in the referral group.

A prior study in a Western population showed that mortality rates in patients with acute myocardial infarction (AMI) were not significantly different between those screened with pre-hospital tele-ECG compared with the controls, both at 30 days and 6 months [12]. However, in higher risk AMI patients, pre-hospital tele-ECG triage has been associated with lower 6-month mortality [12]. In our study, we did not use a control group to compare the performance of pre-hospital tele-ECG since we used the general population in primary care settings as our study population.

While low- and middle-income countries are more likely to consider resource barriers such as high costs, underdeveloped infrastructure, and lack of technical expertise to tackle telemedicine, high-income countries are more likely to consider legal issues surrounding patient privacy and confidentiality, competing health priorities, and perceived lack of demand to be barriers in telemedicine implementation [1]. However, the success of the Makassar Telemedicine Program has shown that implementation of telemedicine (i.e. tele-ECG) in a low–resource setting is feasible and beneficial in the context of early disease detection and selection of patients for referral.

A previous study has suggested that tele-ECG is a practical and cost-effective tool for the diagnosis and monitoring of CVD and accordingly improves the accessibility and quality of care in a rural low-to-middle income population in India [13]. Singh et al. reported that patient satisfaction was ~ 95% [13], while in our study, we accounted for a similar 95% of GP satisfaction in primary care facilities. Another study concluded that pre-hospital tele-ECG is highly appreciated and utilized by the emergency department staff, with 86% indicating excellent satisfaction [14]. In developed countries, both pre- and in-hospital tele-ECG triage significantly shorten door-to-balloon time in patients with acute myocardial infarction and result in higher rates of timely primary percutaneous coronary intervention (PCI) (< 90 min), compared to the control group [12, 15, 16]. Tele-ECG has been relevantly proven to reduce unnecessary hospitalization and incorrect diagnosis in the case of suspected acute CVD [14].

To our knowledge, the present study is among the first to explore the implementation of telemedicine programs in South-East Asia and couples the program performance to patient outcomes. During follow-up, we had to cope with the unorganized and incomplete patient data at primary care centers (Puskesmas). Follow-up would be far easier if all Puskesmas kept standardized and reliable medical records. In the future, we hope that first, primary care records should be available in the form of an electronic database to ease integration and communication with hospitals. Second, patient and doctor engagement and long-term planning for primary or secondary prevention should be managed better. Each patient should have a solitary permanent record for all check-ups and consultations. Third, patients who are eligible and willing to participate in a research study should provide a copy of an official ID card (e.g. residence permit or kartu tanda penduduk; or driver’s license or surat ijin mengemudi) to ensure that follow-up and data acquisition from hospital or primary care centers could be performed efficiently.

During the entire follow-up (14 ± 6.6 months), seven (1.4%) patients died and 96 (19.0%) were hospitalized for CVD. However, due to poor medical records in Puskesmas in Indonesia, particularly in Makassar, there is no primary care database available. Therefore, any comparison in terms of cardiovascular mortality or hospitalization is not possible. This study has other potential limitations. First, before this tele-ECG program, the ECG assessment did not exist in most primary care centers in Makassar, and hence comparison regarding the waiting time, performance, or other evaluation tasks before and after the implementation of tele-ECG was also unfeasible.

Second, we assumed that there were more undiagnosed and undetected patients with diabetes in our study population, meaning that we might have underestimated the rate of CVD risk factors. The CVD risk profiles could be even worse than we observed. However, this limitation is unlikely to have biased our main results. We suggest that the Indonesian government should be more serious about combating CVD risk factor burden in this country.

Considering that atherosclerotic CVD and diabetes are the leading causes of mortality and morbidity in Indonesia, detection and screening of diabetes and dyslipidemia should be available and affordable at the primary care level [2, 4]. Third, the effectiveness of the tele-ECG program can only be estimated, as data collection did not allow for reliable calculation of false-negative and false-positive ratios. Last, one has to be aware of the fact that the study design required the GPs to send all ECG assessments to the database center, and thus, we possibly overestimated the report of GPs reasoning on making the consultation.

## Conclusions

In conclusion, in a less-developed country, tele-ECG is feasible and affordable to assist primary care GPs in conducting a quick triage to recognize life-threatening CVD events based on expert advice. The use of tele-ECG in this resource-limited setting indicates a higher rate of early hospitalization for the indicated patients.

## Supplementary Information

**Additional file 1.**

## Data Availability

All data generated or analyzed during this study are included in this published article or uploaded as supplementary materials. No additional data are available. The dataset is available from the corresponding author (myaqanitha@gmail.com) upon reasonable request.

## Abbreviations

ECG: Electrocardiography
GPs: General practitioners
CVD: Cardiovascular disease
CAD: Coronary artery disease
DALYs: Disability-adjusted life-years
Puskesmas: Pusat kesehatan masyarakat
SD: Standard deviation
AMI: Acute myocardial infarction
PCI: Percutaneous coronary intervention
