# Supplementary figures and images for: Tele-ECG consulting and outcomes on primary care patients in a low-to-middle income population: the first experience from Makassar telemedicine program, Indonesia
Source: BMC Fam Pract. 2020 Nov 30;21:247. doi: 10.1186/s12875-020-01325-4 (PMC7702690; doi:10.1186/s12875-020-01325-4)

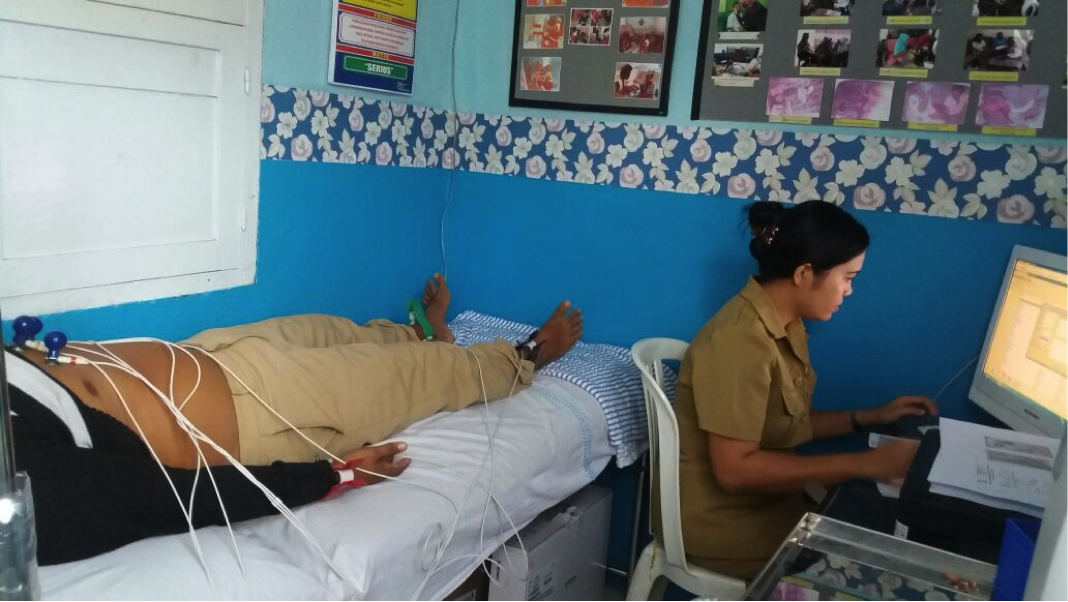

Supplement: Supplementary file 1 — Additional file 1. [file 12875_2020_1325_MOESM1_ESM.zip › BMC FP_Fig. S1 (a)R1.png]

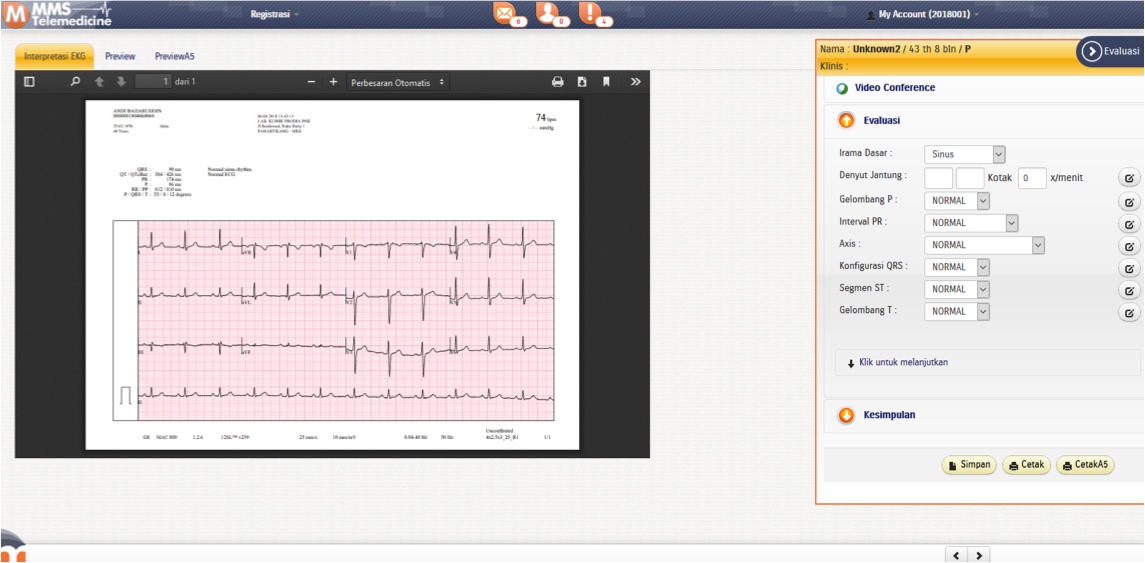

Supplement: Supplementary file 1 — Additional file 1. [file 12875_2020_1325_MOESM1_ESM.zip › BMC FP_Fig. S1(b)R1.png]
